# Supplementary material for: Analysis of the Biomarkers for Neurodegenerative Diseases in Aged Progranulin Deficient Mice
Source: Int J Mol Sci. 2022 Jan 6;23(2):629. doi: 10.3390/ijms23020629 (PMC8775568; doi:10.3390/ijms23020629)
Supplement: Supplementary file 1 [file ijms-23-00629-s001.zip › ijms-1509735-supplementary.pdf]

Analysis of the biomarkers for neurodegenerative diseases in aged-progranulin deficient mice

Xiangli Zhao, Sadaf Hasan, Benjamin Liou, Yi Lin, Ying Sun, Chuan-ju Liu*

*To whom correspondence should be addressed: Rm 1608, LOH, 301 East 17th Street, New York, NY 10003. Tel: 212-598-6103; Fax: 212-598-6096; Email: chuanju.liu@nyumc.org

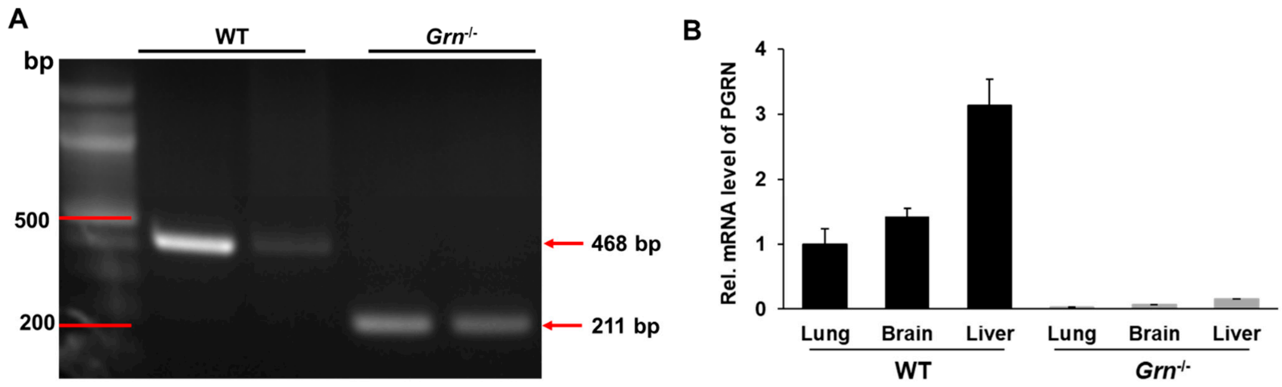

Supplementary Figure S1. PGRN-deficient mice (*Grn*$^{-/-}$). (A) Genotyping of *Grn*$^{-/-}$ mice. (B) Lung, brain and liver tissues were collected from 2-month-old WT mice or *Grn*$^{-/-}$ mice. After homogenization, RNA isolation, and reverse transcription, the cDNA of the indicated samples was used to perform RT-PCR.

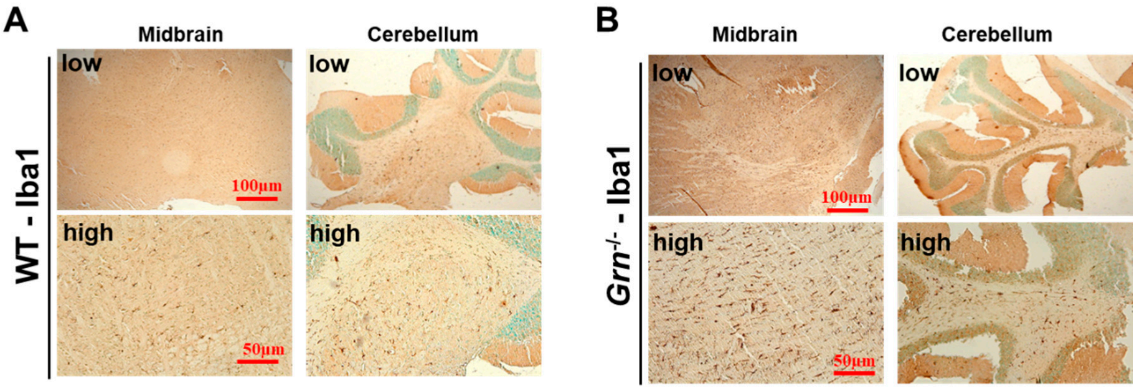

Supplementary Figure S2. Ablation of PGRN led to the activation of microglia in aged mice. Brain sections from 12 m-old WT and *Grn*$^{-/-}$ mice were stained with anti-Iba1 antibody. The accumulation of Iba1-positive cells was analyzed in different regions of the brain, including the midbrain and cerebellum, using Immunohistochemistry. (A) Representative images of two different brain regions from WT mice. (B) Representative images of two different brain regions from *Grn*$^{-/-}$ mice.

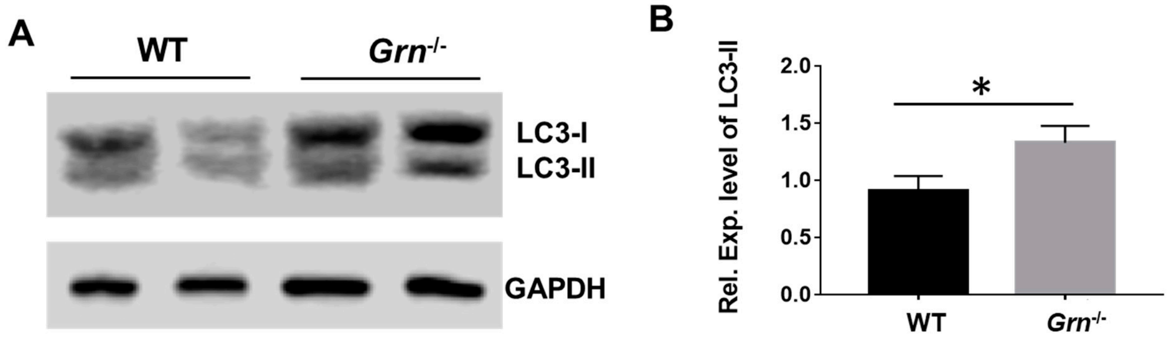

Supplementary Figure S3. Autophagy was defective in aged *Grn*$^{-/-}$ mice. Brain tissues were collected from 12-month-old WT mice or *Grn*$^{-/-}$ mice, and then homogenized and lysed in RIPA lysis buffer. The tissue lysates were used to perform Western blot. (A) Western blot analysis of LC3-II in the 12-month-brain tissue of WT and *Grn*$^{-/-}$ mice. (D) Statistic analysis of A. $n = 3$. Data presented as mean ± SD. *p<0.05.
